# Eating disorders and psychiatric comorbidity among first-year university students in Sweden: Prevalence and risk factors

**DOI:** 10.1186/s40337-025-01230-0

**Published:** 2025-03-20

**Authors:** Catharina Strid, Petra Lindfors, Claes Andersson, Anne H. Berman

**Affiliations:** 1 Department of Psychology, Lund University, Lund, Sweden; 2 Department of Psychology, Stockholm University, Stockholm, Sweden; 3 Department of Criminology, Malmö University, Malmö, Sweden; 4 Department of Psychology, Uppsala University, Uppsala, Sweden

**Keywords:** Eating disorders, Co-morbidity, University students, Mental health, Prevalence

## Abstract

**Background:**

This study explored eating disorders (ED) prevalences, comorbidity of ED with other mental disorders, and risk factors for ED among university students. ED included binge eating disorder (BED), bulimia nervosa (BN), or other specified feeding and eating disorders (OSFED).

**Methods:**

A total of 3425 first-year university students in Sweden completed an online survey covering a range of criteria for psychiatric diagnoses, within the World Mental Health International College Student (WMH-ICS) initiative. Pearson’s χ^2^ -tests were used to compare algorithm-based diagnostic prevalences for eating disorders and other comorbid psychiatric disorders between three groups: students with ED with or without other comorbid psychiatric disorders (A), students with psychiatric disorders but no ED comorbidity (B), and students with no psychiatric disorders (C). Multinomial logistic regression was used to calculate between-group comparisons of odds ratios for independent risk factors, where group B served as the reference group for comparisons with groups A and C.

**Results:**

Of the total sample, 75% had at least one psychiatric disorder and 28% had at least one lifetime ED diagnosis. Students with ED (group A) reported higher prevalences for comorbid anxiety disorders, depression, suicidal behavior, and non-suicidal self-injury compared to students with psychiatric disorders but no ED (group B). Group A participants exhibited a higher risk of hazardous drinking, were more likely to have received medical treatment, and to identify as bisexual. Compared to group B, students with no psychiatric disorders (group C) were more likely to report better mental and physical health, but less likely to engage in hazardous drinking, and to have sought mental health treatment.

**Conclusions:**

A large proportion of students with ED had additional psychiatric disorders, indicating that individuals with ED suffer from multiple mental health problems. It is crucial that student health services acquire competency to offer effective ED assessment and treatment.

**Supplementary Information:**

The online version contains supplementary material available at 10.1186/s40337-025-01230-0.

## Abstract (plain language summary)

This study, as part of an international initiative on the mental health of college students, aimed to explore the occurrence of eating disorders and related risk factors among first year university students in Sweden. In 2020–2021, 3425 Swedish first-year students answered an online survey about their mental health. The results for lifetime disorders showed that 75% of the students had at least one psychiatric disorder, while 28% had at least one eating disorder, and 17% had no psychiatric disorders. Students with eating disorders reported a high co-morbidity with anxiety, depression, suicidal behavior, self-injury, and risk for harmful drinking. In contrast, students with no psychiatric disorders were more likely to report good mental and physical health and less likely to report harmful drinking. The finding that, only 17% reported no psychiatric problems, while as many as 28% reported eating disorders may be related to the study having been conducted during the COVID-19 pandemic when the reporting of eating disorders increased. Nevertheless, the results show that students with eating disorders suffer from other psychiatric problems to a large extent. This underscores the need of student health services to provide targeted treatment and support.

**Supplementary Information:**

The online version contains supplementary material available at 10.1186/s40337-025-01230-0.

## Background

Systematic literature reviews show high prevalence estimates for eating disorders (ED) in the population [1, 2]. The lifetime estimated prevalence of ED in the Nordic countries ranges from 0.1 to 6.5% in men and 7% to 17.9% in women [3, 4]. In a study including Europe and the United States (US), the estimated lifetime prevalence for ED ranged between 0.8 to 6.5% in men and 3.3% to 18.6% in women [5]. The risk for ED is thus several times higher in women. The incidence of ED is furthermore highest during adolescence and young adulthood [4, 6, 7]. This is a period in life when individuals are forming their identity and developing autonomy and social roles, as they move into new study or work contexts [8]. At university, students face several challenges during this vulnerable phase of life and are at heightened risk of developing common mental health disorders. This is reflected in studies showing high prevalence rates of anxiety and depression among students, with an estimated lifetime prevalence between 19 and 48%, and 80–90% overlap with past 12-month prevalences [9, 10]. As increasing numbers of young adults enter into higher education, this context constitutes a significant arena for preventive interventions, treatment and support to students with different mental health problems [11].

Students commonly report a variety of ED symptoms such as binge eating, calorie-restrictive dieting or other maladaptive eating habits, and overall prevalence estimates range from 3 to 20% [12–14]. Most studies on ED among students have focused on prevalence, comorbidity with other mental health disorders, and risk factors for ED. Findings including 4889 students in the WHO World Mental Health International College Student (WMH-ICS) initiative in Belgium showed a 12-month DSM-IV prevalence of binge eating disorder (BED) at 7.3% and prevalence of other specified feeding and eating disorder (OSFED) at 1.0%. BED and OSFED were associated with several mental health problems such as non-suicidal self-injury (NSSI), post-traumatic stress, and suicidal ideation and also with a high risk of academic failure [15]. Other studies have shown that individuals with ED often report symptoms of anxiety and depression, with anxiety being the most frequent comorbidity diagnosis which, in turn, has been related to a more severe ED [13, 16, 17]. Also, eating problems have been associated with problematic alcohol use, with studies showing comorbidity between ED and alcohol use disorder [18, 19]. Taken together, these research findings provide a rationale to focus specifically on EDs and associated risk factors.

The key clinical problem in ED diagnosis involves abnormal eating behaviors and problems with body shape and weight. ED severity can range from mild difficulties to severe problems, and ED causes both physical and psychological difficulties and a high mortality risk [20]. Treatment accessibility for ED is low, and several barriers have been identified for individuals with ED in seeking and accessing treatment [21–23]. Digital interventions can reduce the treatment gap by lowering the threshold for treatment access [24], and digital interventions for ED have been suggested to yield positive outcomes [25]. Preparing for a future scenario where existing effective digital interventions for ED could be adapted to the needs of university students and scaled up for more accessible delivery, it is necessary to first clarify the ED prevalence in this target group. It should be noted that there is a difference between diagnosed ED and disordered eating, where the latter refers to subclinical problems not meeting the criteria for a specific diagnosis. Disordered eating can also co-occur with other mental health problems and has a negative impact on health-related quality of life [19, 26, 27]. Students with a minority sexual orientation are especially vulnerable to disordered eating behaviors [28–30]. This study is a part of a larger initiative on students’ mental health [31]. Focusing on diagnosed ED is a first important step towards the development of accessible treatment to be provided by students’ mental health services. Importantly, this approach could be beneficial for students with disordered eating as well.

This study thus aimed to explore the prevalence of diagnosed ED, its comorbidity with other mental disorders, and risk factors for ED diagnosis among first-year university students in Sweden. To our knowledge, no previous research has been reported on ED among the university student group in Sweden. Specific research questions concerned the prevalence of ED diagnoses according to the DSM-IV, with a specific focus on BED and OSFED, among students with and without comorbid psychiatric disorders. Diagnoses and risk factors were compared between three groups: students reporting ED with or without other comorbid psychiatric disorders, students reporting psychiatric disorders but no ED comorbidity, and students reporting no psychiatric disorders.

## Methods

### Participants

This study was conducted within the WHO WMH-ICS initiative in Sweden, ongoing since 2020 [31, 32]. The sample included first-year students from six universities in Sweden during the 2020–21 academic year, surveyed in two separate cohorts from the fall and spring terms, respectively. This period coincided with the then-ongoing COVID-19 pandemic, which initially entailed comprehensive restrictions of onsite teaching activities at all Swedish universities. Of a total of 28 809 students invited to respond to the survey, 5 893 (20%) consented to participate of whom 719 (12%) dropped out, 1749 (30%) did not complete the survey and 3425 (58%) completed the survey, corresponding to about 12% of the invited students. Among consenting respondents, a comparison between those not completing the survey and the survey completion sample showed that the latter were older, included more women, and had poorer self-rated mental and physical health, (see Supplementary file 1).

### Procedure

First-year students enrolled in educational programs within the arts, humanities, medicine, social sciences, and engineering were contacted through e-mail lists provided by seven institutions of higher education participating in the WMH-ICS project in Sweden. Students were invited to take part in an online survey. All participants granted their informed consent online before gaining access to the survey. Analyses were conducted on an anonymous data set and results are reported on the group level. The procedures were reviewed by the Swedish Ethical Review Authority (Ref. No. 2020‐01465, approved May 11, 2020).

### Measures

#### Survey

The WMH-ICS survey [9, 10] used in this study consists of 11 sections, covering the following areas: a) your background, b) your health, c) attention and concentration, d) emotional problems, e) alcohol and drugs, f) self-harm, g) seeking treatment, h) childhood background, i) recent experiences, j) sexuality, and k) concept of self.

#### Variables included in this study

Demographic variables included in this study were age, sex at birth, gender identity, civil status, and student status. Based on earlier research, additional survey variables considered as risk factors for ED and other psychiatric disorders were included in this study: mental and physical health, hazardous drinking, sexual orientation and lifetime treatment-seeking behavior. These additional variables were measured as follows:

Mental health was measured by asking “How would you rate your overall mental health?” and physical health by asking “How would you rate your overall physical health?”. The responses were coded from 1 (Excellent) to 5 (Poor). The answers were dichotomized into better; including excellent, very good, and good (coded as 1) and worse; including fair, and poor (coded as 2) health in the analyses.

Hazardous drinking was measured using the short form of Alcohol Use Disorder Identification Test, AUDIT-C [33] with three questions assessing alcohol consumption: 1. “How often do you have a drink containing alcohol?” 2. “How many drinks containing alcohol do you have on a typical day when you are drinking?” 3. “How often do you have 4 (women)/5 (men) or more drinks on one occasion?”. As recommended in the Swedish guidelines [34], hazardous drinking was defined according to standard cut-off scores used in Sweden (≥ 6 for men and ≥ 5 for women).

Sexual orientation was indicated by responding to one question “What is your sexual orientation?” Response alternatives were as follows: a) Heterosexual or straight, b) gay or lesbian, c) bisexual, d) asexual, e) not sure and f) other.

Past lifetime treatment included three different specified forms of treatment with the question “Did you ever in your life receive psychological counseling, medication, or some other type of treatment for an emotional or substance use problem?”.

The diagnoses in WMH-ICS were calculated based on participant responses to questions deriving from the Composite International Diagnostic Interview Screening Scales (CIDI-SC). The survey questions include items with multiple response alternatives and generate DSM-IV diagnoses; for details see WMH-ICS publications [9, 10, 35]. Diagnostic variables included in the analyses were ED (described in detail below), generalized anxiety disorder, major depressive disorder, intermittent explosive disorder, social anxiety, and drug use disorder; as well as problem areas such as suicidal ideation and, non-suicidal self‐injury (NSSI). All diagnoses were assessed for lifetime and 12-month prevalences.

The lifetime prevalence of ED included measures of BED, Bulimia Nervosa (BN), and OFSED. BED was assessed by asking “Did you ever in your life have times lasting 3 months or longer when you had eating binges at least once a week; that is, your eating was out of control, and you ate a very large amount of food over a short period of time (2 h or less)**?** BN was assessed by the question, “Did you ever in your life have times lasting 3 months or longer when you made yourself vomit, took laxatives, or did other things to avoid gaining weight after binge eating?” OFSED was assessed by the question “Did you ever in your life have times lasting 3 months or longer when you made yourself vomit, took laxatives, or did other things to avoid gaining weight?” The past 12-month prevalence of BED was captured by asking how many months respondents had engaged in binge eating behaviors at least once a week. For BN, this was assessed by asking, “About how many months in the past 12 did you make yourself vomit, take laxatives, or do other things to avoid gaining weight after binge eating?” and for OFSED by asking “About how many months in the past 12 did you make yourself vomit, take laxatives, or do other things to avoid gaining weight?”.

Statistical analyses.

Data were analyzed with IBM SPSS statistics version 27 software. Three analytical procedures were conducted, including descriptive statistics, comparisons between diagnostic sub-groups in the data set, and risk factors predicting sub-group categorization. Descriptive statistics (means or percentages) were calculated for the prevalence of socio-demographic variables, ED diagnoses, and comorbidities with other psychiatric disorders in the total group, and separately for women and men. Then the data set was divided into diagnostic subgroups as follows: students reporting ED with or without psychiatric comorbidity (group A; the group of students reporting ED only was small n = 77, but retained in group A since students with ED are likely to need treatment even without comorbid disorders); students reporting psychiatric disorders without ED comorbidity (group B); and students reporting no psychiatric disorders (group C). Pearson’s χ^2^ -tests were used to compare between-group prevalences of psychiatric diagnoses and the problem areas of suicidal ideation and NSSI. Multinomial logistic regression, with a confidence level of 95%, was used to calculate between-group comparisons of odds ratios for independent risk factors. Five of the independent variables were categorical. Three of these were binary: self-rated mental and physical health, each dichotomized as better or worse; alcohol use, dichotomized as hazardous or non-hazardous drinking. Two of the independent variables (sexual orientation and lifetime treatment) included more than two categories. Correlations were estimated to assess the best model fit. The dependent variable in this analysis was sub-group categorization into groups A, B, or C. The effects of age and sex at birth, respectively, were calculated in a separate sensitivity analysis (see Supplementary file 2).

## Results

The total sample of 3425 first-year students included 2440 women and 979 men, with a mean age of 24 years (SD 5.5), and the majority studying full-time (Table 1). A higher percentage of men were single and heterosexual, where a bisexual orientation was more common in women. As many as 75% of the total sample self-reported having experienced at least one lifetime psychiatric disorder, and 28% reported at least one ED; ED was more common in women than men.

**Table 1:** Descriptive statistics for first-year students in Sweden (*n* = 3425), total and by sex at birth

	Total sample (*n* = 3425)	Men (*n* = 979)	Women (*n* = 2440)
Age, years M (± Sd)	24 (5.5)	23.8 (5.3)	24.2 (5.6)
Gender identity %			
Men	29	98	0.5
Women	71	1	98
Other	2	1	1.5
Civil status %			
Married	28	20	30
Living together	5	5	5
In a relationship	15	13	16
Dating	8	8	9
Single	43	54	40
Student status %			
Full-time	95	95	95
Part-time	3	3	3
Other^a^	2	2	2
Sexual orientation %			
Heterosexual	75	84	71
Homosexual	3	3.5	3
Bisexual	13	6.5	15
Asexual	1	1	1
Do not know	5.5	3	6.5
Other^b^	2.5	1.5	3
Life-time mental health %			
ED^c^ only	2	1	3
ED with psychiatric comorbidity	26	13	32
Psychiatric disorders without ED comorbidity	47	54	44
No psychiatric disorders	17	23	5
Missing	7	8	7
Last 12-month mental health %			
ED only	4	2	4
ED with psychiatric comorbidity	18	10	20
Psychiatric disorders without ED comorbidity	40	40	40
No psychiatric disorders	31	40	28
Missing	7	8	7
Alcohol consumption %			
Abstainers	18	16	18
Drinkers	82	84	82
Hazardous (% of drinkers)	37	33	33
Treatment, lifetime %			
Psychological	43	32	48
Medication	24	16	27
Other type of treatment	8	4.5	10

a. Other student status included free-standing courses and study status that was not subsumed under full-time or part-time and specified in text boxes, e.g. studying two educational programs at once

b. Other sexual orientations were further specified in text boxes e. g. pan sexual

c. ED – eating disorder

Table 2 shows lifetime and 12-month prevalence rates for the different ED diagnoses. In the total sample, ED prevalence varied by ED type. BED was the most common problem, with a lifetime prevalence of 21% and past year prevalence of 13%. Of the 13% reporting BED during the last 12 months, 68% reported that BED occurred at least once a week for 1 to 6 months, and 13% reported that BED occurred every week for the last 12 months. The prevalence for BN was 7% for lifetime and 4% for 12-month prevalence. Of those with BN, 28% reported experiencing problems once a week or more often during the past 12 months. The lifetime prevalence for OFSED was 15%, while the 12-month prevalence was 5%. Of these, 10% reported experiencing weekly problems during the past 12 months.

**Table 2:** Prevalence rates of Binge eating disorder (BED), Bulimia Nervosa (BN), and Other specified feeding or eating disorder (OFSED) among first-year students in Sweden (n = 3425)

	BED	BN	OFSED
Lifetime, %	21	7	15
Past 12 months, %	13	4	5
Months/year with symptoms %			
1–6 months	68	50	71
7–11 months	18	22	19
12 months	13	28	10
Missing %	6	7	4

Table 3 shows lifetime psychiatric disorders among students reporting ED with or without psychiatric comorbidity (group A, n = 979), students reporting psychiatric disorders without ED comorbidity (group B, n = 1604), and students reporting no psychiatric disorders (group C, n = 842). Comparisons of lifetime psychiatric disorders in groups A and B showed that students with ED reported significantly higher prevalence rates for anxiety disorders, depression, suicidal behavior, and non-suicidal self-injury behavior. The two groups did not differ in intermittent explosive disorder and problematic drug use.

**Table 3:** Prevalence of lifetime psychiatric disorders in two student groups: A) eating disorders with or without psychiatric comorbidity; and B) psychiatric disorders without eating disorders comorbidity

Diagnosis %	Group A N = 979	Group B N = 1604	Chi-2 (*df* = *1*)	*p*
Generalized anxiety disorder^a^	49	33	62.38	< 0.001
Major depressive episode	72	57	56.62	< 0.001
Intermittent explosive disorder	25	22	2.91	0.088
Social anxiety	38	31	12.04	< 0.001
Drug abuse	14	13	0.75	0.388
Suicidal ideation	70	64	7.90	0.005
Non-suicidal self-injury	50	31	96.54	< 0.001

^a^Group A *n* = 924 Group B *n* = 1487

Table 4 shows the results of the multinomial regression, where group B, students with psychiatric disorders but no ED comorbidity, was set as the reference group. The overall model was statistically significant, χ^2^ = 772.868 (24), p < 0.001, with a Nagelkerke R-squared value of 0.255, indicating that the model explained 26% of the variance in the dependent variable, i.e., categorization in group A, B or C with differing prevalences of psychiatric disorders. The model correctly classified 54% of the cases. Students with ED, with or without psychiatric comorbidity (A), rated their mental health as poorer and had a higher risk of hazardous and harmful drinking compared to students in the reference group (B), who had psychiatric disorders without ED comorbidity. Students in Group A were also more likely to identify as bisexual compared to the reference group. Furthermore, those in Group A were more likely to have used medical or alternative treatment outside the public healthcare system during their lifetime compared to the reference group. It should be noted that there were no statistically significant differences between groups A and B in self-rated physical health, sexual minority status (aside from bisexuality), and lifetime of psychological treatment.

**Table 4:** Results of multinomial logistic regression with three groups: A) eating disorders (ED) with or without psychiatric comorbidity (*n* = 979), B) psychiatric disorders without eating disorders comorbidity (*n* = 1604), and C) no psychiatric disorders (*n* = 842). Group B is the reference group in the model

	b(SE)	Odds ratio	CI [95%] Lower	Upper
Group A: ED with or without psychiatric comorbidity^a^				
Intercept	−0.709(0.112)			
Physical health, self-rated	−0.154(0.100)	0.857	0.705	1.042
Mental health, self-rated	−0.182(0.089)*	.833	0.700	0.993
Hazardous drinking	0.399(0.092)***	1.490	1.243	1.785
Sexual orientation				
Gay or Lesbian	−0.163(0.229)	1.177	0.751	1.844
Bisexual	0.335(0.118)**	1.399	1.109	1.763
Asexual	0.583(0.339)	1.792	0.923	3.479
Do not know	0.146(0.173)	1.157	0.825	1.622
Other	0.394(249)	1.484	0.911	2.416
Treatment, lifetime				
Psychological	0.117(0.102)	1.124	0.921	1.370
Medical	0.340(0.110)**	1.405	1.133	1.742
Other	0.491(0.146)***	1.634	1.228	2.174
Group C: No psychiatric disorders				
Intercept	−2.286(0.214)***			
Physical health, self-rated	0.774(0.182)***	2.169	1.518	3.10
Mental health, self-rated	1.6171(0.160)***	5.317	3.886	7.276
Hazardous drinking	−0.394(0.129)**	0.675	0.524	0.868
Sexual orientation				
Gay or Lesbian	−0.559(0.433)	0.572	0.245	1.336
Bisexual	−0.800(0.240)***	0.449	0.281	0.719
Asexual	−1.242(0.774)	0.289	0.063	1.317
Do not know	−0.737(0.310)*	0.479	0.261	0.879
Other	−0.699(0.474)	0.497	0.196	1.258
Treatment, ever				
Psychological	−1.302(0.163)***	0.272	0.198	0.374
Medical	−0.836(0.260)**	0.433	0.260	0.722
Other	−0.460(0.292)	0.631	0.356	1.119

^a^Students with only ED were included in this group

*p < 0.05, **p < 0.01, ***p < 0.001

Compared to the reference group, students with no psychiatric disorders (group C) were more likely to report better self-rated physical and mental health, less likely to engage in hazardous drinking, less likely to identify as bisexual and, to some extent, to be unsure about their sexual orientation. Furthermore, they were less likely to have sought psychological and medical treatment. Notably, there were no significant differences between groups C and B in having sought treatment outside the ordinary healthcare system, as well as in sexual minority identification, apart from bisexuality and lack of certainty regarding sexual orientation.

The results from the sensitivity analysis showed that sex at birth reduced the risk for ED among bisexual students since most of the respondents identifying as bisexual were women. Concerning the effect of age, the analysis showed that the students with no psychiatric disorders (group C) were, on average, older than the students with psychiatric problems, (see Supplementary file 2).

## Discussion

The present findings show that students who responded to the WHO-WMH-ICS survey in Sweden reported several ED problems. Specifically, the lifetime prevalence of ED was higher than in previous reports of student cohorts, 28% compared to between 3 and 20% in different Western and non-Western countries [12–14]. This may relate to the large proportion of women (71%) in the present study sample. Still, this gender distribution can be considered reasonably representative of the undergraduate student target population in Sweden [36]. Moreover, the average age of the students in our sample (24 years) was comparable to that among students at Swedish universities [36]. However, students in Sweden are, as our study sample, generally older than in international studies investigating eating disorders in students [14], a circumstance that may explain the observed discrepancies in lifetime prevalences.

The higher lifetime prevalence of ED in the present student cohort, compared to prior research on students, is somewhat challenging to interpret. Still, the high proportion of women (71%) and mean age (24 years) may be part of the explanation, as female gender and age correlate positively with the life-time prevalence of mental disorders [10, 37]. Women also report ED problems to a higher degree then men [1, 38]. However, the finding may also be related to the timing of the data collection; during the COVID-19 pandemic, all teaching was moved online. Reports of common mental health problems such as anxiety, depression, and ED among students increased during this period [39–41], in spite of high compliance with recommendations to limit viral contagion [36]. BED was the most commonly reported ED diagnosis, with a lifetime prevalence of 21% and a 12-month prevalence of 13%. Still, it should be noted that the behavioral frequency of binge eating was low occurring at just over 10% at least once a week throughout the year. However, the 12-month prevalence was somewhat higher: 13%, compared to 7% in a previous study using the same questions [15], which did not include reports on the behavioral frequency of binge eating per month during the last 12 months, making comparisons more difficult. Overall, the 12-month prevalence of BED and OFSED in the present sample showed that some students have frequent and long-lasting problems and seem to fulfill DSM-5 diagnostic criteria, given that the behavioral frequency question in the WMH-ICS survey asked about binging and vomiting behaviors at least once a week, corresponding to DSM-5 criteria, whereas DSM-IV criteria required reporting of such behaviors at least twice a week. In contrast, other students seem to have disordered eating behaviors less frequently, indicating more of a sub-clinical eating problem. Among students with BN, almost one-third reported frequent ED behaviors during the past 12 months and seem to suffer from more severe problems that may require treatment in specialized care. Importantly, this analysis of eating disorders among students provides a basis for student healthcare providers to plan for tailored support and interventions that target both clinical and sub-clinical problems relating to EDs.

A large proportion of students with ED reported other psychiatric disorders, such as depression and anxiety, but also suicidal ideation and NSSI. This indicates, in line with previous studies [16, 17], that individuals with ED suffer from several mental health problems. For instance, a US study [42] reported a strong association between disordered eating and NSSI among university students with a high risk of suicidal behavior. Also, a large systematic review and meta-analysis, including studies from 11 countries, found a 27.3% life-time prevalence of NSSI in patients with ED [43]. Moreover, the NSSI prevalence increased with an increasing number of suicide attempts. With 50% of the students with ED in the present sample reporting NSSI, and as many as 70% reporting suicidal ideation, our findings corroborate the association between ED, NSSI, and suicidal behavior. It is notable that the figures were significantly higher than those for students with psychiatric disorders without ED comorbidity (group B).

Overall, the present findings underscore the potential severity of ED conditions, with several difficult symptomatic behaviors such as anxiety, NSSI, suicidal ideation, and depression. From a treatment perspective, possible transdiagnostic mechanisms such as impulsivity, affective dysregulation, and alexithymia are involved in these behaviors [44]. This makes it important for student healthcare providers to broaden their assessment range and, in addition to targeting eating problems, to also address anxiety, impulsive behaviors such as NSSI, and suicidal ideation in treatment programs. However, with ED often going undetected [45], ED symptoms should also be assessed among students seeking help for other conditions such as anxiety or depression.

An additional finding related to students with bisexual orientation having a higher risk for ED. This aligns with previous studies, including Calszo and colleagues, who found that girls with a minority sexual orientation had twice the risk of elevated binge eating and purging by mid-adolescence, compared to heterosexual girls [28]. Further supporting this finding, a recent systematic review by Cao and colleagues concluded that youth with a minority sexual orientation are more vulnerable to ED [46].

Hazardous alcohol use was common in this student cohort and, in line with earlier studies [19, 47], those with ED were at greater risk for hazardous drinking compared to students with other psychiatric disorders without ED comorbidity. Heavy drinking is common among students and is often a central and accepted part of student social life [48]. This may complicate the identification of harmful drinking in students with ED or in detecting ED in students with drinking problems.

As expected, students reporting no psychiatric disorders rated their mental and physical health as better to a much larger extent than did the ED and psychiatric disorder groups. They had a lower risk for hazardous drinking and were also less likely to report lifetime medication or psychological treatment.

Students with ED reported lifetime treatment with medication more often than students with psychiatric disorders without ED comorbidity. Still, self-reports of past experiences of psychological treatment did not differ between the two groups. This finding might reflect unmet healthcare needs and a specific lack of access to psychological treatment for common mental health problems [9, 23, 49, 50]. Current access to treatment for eating disorders is restricted to specialist healthcare services, but the pathways to healthcare are complicated and long [51]. Also, treatment-seeking barriers are pronounced, delaying access to appropriate ED treatment by several years [52]. Cognitive behavioral therapy has shown positive results for treating ED, also when Internet-delivered [53, 54]. It is conceivable that university healthcare services might more easily offer internet-based treatment, with counselor support primarily via text-based messaging, for students with differing types of eating problems. This treatment step would require fewer resources than face-to-face treatment and could reach more students [55]. Student mental health services in Sweden are legally obliged to offer preventive care. Screening for ED among patients seeking help for other problems and offering internet-based treatment for students reporting disordered eating could, as a start, be rationalized as an instance of indicated prevention with the potential of shortening the pathway to specialist care and, eventually, lowering ED prevalences in student populations.

### Strengths and limitations

Restricted to first-year students, a particularly vulnerable group, the present study sample was relatively large. The timing of the data collection during COVID-19 and the possibility that the study attracted students with great experience of psychological problems may have influenced the findings by contributing to an overestimation of the prevalence of ED symptoms corresponding to estimated diagnoses. Also, the wording of survey questions, particularly the question asking about BED, may have yielded an overreporting of binge eating symptoms. Regardless, EDs may be largely unaddressed throughout the university years. Notably, the symptom levels described in our study included both clinical and sub-clinical levels of ED. Both groups are likely in need of preventive care or targeted treatment.

An obvious limitation relates to the assessment of psychiatric symptoms, including symptoms of ED, through self-reports. Nonetheless, previous studies show that self-report measures with good criterion validity have good to excellent concordance with structured interviews [56]. Also, resources are unavailable to carry out thousands of structured interviews, meaning that self-reports provide a feasible alternative. Still, the finding that only 17% of students reported no lifetime psychiatric problems may result from the selection bias and reflect the timing of the data collection. Specifically, during the COVID-19 pandemic, mental health problems may have increased among many vulnerable groups, including first-year students [57].

## Conclusions

Importantly, this is to our knowledge the first study of ED among first-year university students in Sweden. The results showed that students in higher education in Sweden report ED problems, as well as high comorbidity with other psychiatric diagnoses and mental health issues. This underscores the importance for healthcare providers to address disordered eating problems among students seeking help for other problems and to expand the assessment beyond ED symptoms to include anxiety, self-harm, suicidal ideation, and harmful alcohol use as a means of providing effective treatment and support. Moreover, our findings emphasize a need to better understand students with ED and their pathways to care, as well as their experiences of the healthcare system and the extent to which family and friends do or do not provide support. Importantly, the findings highlight a need for low-threshold effective treatments, including, for instance, internet-based cognitive behavioral programs adapted to the student context in higher education.

## Supplementary Information

Additional file1 (DOCX 20 KB)

Additional file2 (DOCX 23 KB)

## Data Availability

The data that support the findings of this study are not openly available due to reasons of sensitivity and are available from the corresponding author upon reasonable request.

## Abbreviations

AUDIT-C: Alcohol use disorder identification test Consumption
BED: Binge eating disorder
BN: Bulimia nervosa
CIDI-CS: Composite international diagnostic interview screening scales
Covid: Corona virus disease
DSM: Diagnostic and statistical manual of mental disorders
ED: Eating disorders
NSSI: Non-suicidal self-injury
OSFED: Other specified feeding and eating disorders
SD: Standard deviation
WMH-ICS: World mental health International college students
WHO: World health organization

## References

[1] Qian J, Wu Y, Liu F, Zhu Y, Jin H, et al. An update on the prevalence of eating disorders in the general population: a systematic review and meta-analysis. Eat Weight Disord Stud Anorex Bulim Obes. 2022;27:415–28. 10.1007/s40519-021-01162-z. 10.1007/s40519-021-01162-z PMC8933366 33834377

[2] Hay P, Aouad P, Le A, Marks P, Maloney D, et al. Epidemiology of eating disorders: population, prevalence, disease burden and quality of life informing public policy in Australia—a rapid review. J Eat Disord. 2023;11:23. 10.1186/s40337-023-00738-7. 36793104 10.1186/s40337-023-00738-7 PMC9933292

[3] Dahlgren Lindvall C, Stedal K, Wisting L. A systematic review of eating disorder prevalence in the Nordic countries: 1994–2016. Nord Psychol. 2018;70:209–27. 10.1080/19012276.2017.1410071.

[4] Silén Y, Sipilä PN, Raevuori A, et al. DSM-5 eating disorders among adolescents and young adults in Finland: a public health concern. Int J Eat Disord. 2020;53:520–31. 10.1002/eat.23236. 31999001 10.1002/eat.23236

[5] Galmiche M, Dechelotte P, Lambert G, Tavolacci MP. Prevalence of eating disorders over the 2000–2018 period: a systematic literature review. Am J Clin Nutr. 2019;2019(109):1402–13. 10.1093/ajcn/nqy342. 10.1093/ajcn/nqy342 31051507

[6] Javaras NK, Runfola CD, Thornton LM, Agerbo E, Birgegard A, et al. Sex- and age-specific incidence of healthcare-register-recorded eating disorders in the complete Swedish 1979–2001 birth cohort. Int J Eat Disord. 2015;48:1070–81. 26769444 10.1002/eat.22467 PMC5028825

[7] Zerwas S, Larsen TJ, Petersen L, Thornton LM, Mortensen PB, et al. The incidence of eating disorders in a Danish register study: associations with suicide risk and mortality. J Psychiatr Res. 2015;65:16e2. 10.1016/j.jpsychires.2015.03.003. 25958083 10.1016/j.jpsychires.2015.03.003 PMC4482129

[8] Arnett JJ. *Emerging Adulthood: The Winding Road from the Late Teens Through the Twenties.* 2014 Oxford University Press. 10.1093/acprof:oso/9780199929382.001.0001

[9] Auerbach RP, Alonso J, Axinn WG, Cuijpers P, Ebert DD, et al. Mental disorders among college students in the world health organization world mental health surveys. Psychol Med. 2016;46:2955–70. 10.1017/S0033291716001665. 27484622 10.1017/S0033291716001665 PMC5129654

[10] Auerbach RP, Mortier P, Bruffaert R, Alonso J, Benjet C, et al. WHO world mental health surveys international college student project: prevalence and distribution of mental disorders. J Abnorm Psychol. 2018;127:623–38. 10.1037/abn0000362. 30211576 10.1037/abn0000362 PMC6193834

[11] OECD. Education at a Glance 2024: OECD Indicators. OECD Publishing, Paris,. 2024. 10.1787/c00cad36-en.

[12] Fekih-Romdhane F, Daher-Nashif S, Alhuwailah AH, Al Gahtani HMS, Hubail SA, et al. The prevalence of feeding and eating disorders symptomology in medical students: an updated systematic review, meta-analysis, and meta-regression. Eat Weight Disord-St. On-line 24 Januari 2022. 10.1007/s40519-021-01351-w 10.1007/s40519-021-01351-w PMC8784279 35067859

[13] Mutiso VN, Ndetei DM, Muia EN, Alietsi RK, Onsinyo L, et al. The prevalance of binge eating disorder and associated psychiatric and substance use disorders in a student population in Kenya – towards a public health approach. BMC Psychiatry. 2022;22:122. 10.1186/s12888-022-03761-1. 35172765 10.1186/s12888-022-03761-1 PMC8848944

[14] Alhaj OA, Fekih-Romdhane F, Sweidan DH, Saif Z, Khudhair MF, Ghazzawi H, Mohammed SH, Nadar SS, Alhajeri MP, Levine HJ. The prevalence and risk factors of screen-based disordered eating among university students: a global systematic review, meta-analysis, and meta-regression. Eating Weight Disord Stud Anorexia Bulimia Obesity. 2022;27(8):3215–43. 10.1007/s40519-022-01452-0. 10.1007/s40519-022-01452-0 PMC9362208 35925546

[15] Serra R, Kiekens G, Vanderlinden J, Vrieze E, Auerbach RP, et al. Binge eating and purging in first-year college students: Prevalence, psychiatric comorbidity, and academic performance. Int J Eat Disorder. 2020;53:339–48. 10.1002/eat.23211. 10.1002/eat.23211 31868255

[16] Ulfvebrand S, Birgegård A, Norring C, Högdahl L, von Hausswolff-Juhlin Y. Psychiatric comorbidity in women and men with eating disorders results from a large clinical database. Psychiatr Res. 2015;230:294–9. 10.1016/j.psychres.2015.09.008. 10.1016/j.psychres.2015.09.008 26416590

[17] Elran-Barak R, Goldschmidt AB. Differences in severity of eating disorder symptoms between adults with depression and adults with anxiety. Eat Weight Disord-St. 2021;26:1409–16. 10.1007/s40519-020-00947-y. 10.1007/s40519-020-00947-y PMC7906044 32592138

[18] Gadalla T, Piran N. Co-occurrence of eating disorders and alcohol use disorders in women: a meta analysis. Arch Womens Ment Health. 2007;10:133–40. 10.1007/s00737-007-0184-x. 17533558 10.1007/s00737-007-0184-x

[19] Mustelin L, Latvala A, Raevuori A, Rose RJ, Kaprio J, et al. Risky drinking behaviors among women with eating disorders—a longitudinal community-based study. Int J Eat Disorder. 2016;49(6):563–71. 10.1002/eat.22526. 10.1002/eat.22526 27038220

[20] Smink FRE, van Hoeken D, Hoek HW. Epidemiology of eating disorders: incidence, prevalence and mortality rates. Curr Psychiatry Rep. 2012;14:406–14. 10.1007/s11920-012-0282-y. 22644309 10.1007/s11920-012-0282-y PMC3409365

[21] Thompson C, Park S. Barriers to access and utilization of eating disorder treatment among women. Arch Womens Ment Health. 2016;19:753–60. 10.1007/s00737-016-0618-4. 26971265 10.1007/s00737-016-0618-4

[22] Lipson SK, Jones MJ, Taylor CB, Wilfley DE, Eichen DM, Fitzsimmons-Craft EE, Eisenberg D. Understanding and promoting treatment-seeking for eating disorders and body image concerns on college campuses through online screening, prevention and intervention. Eat Behav. 2017;25:68–73. 10.1016/j.eatbeh.2016.03.020. 27117825 10.1016/j.eatbeh.2016.03.020 PMC5403617

[23] Byrom NC, Batchelor R, Warner H, Stevenson A. Seeking support for an eating disorder: a qualitative analysis of the university student experience—accessibility of support for students. J Eat Disorder. 2022;10(e10):33. 10.1186/s40337-022-00562-5. 10.1186/s40337-022-00562-5 PMC8903729 35256006

[24] Kazdin AE. Addressing the treatment gap: a key challenge for extending evidence-based psychosocial interventions. Behav Res Ther. 2017;88:7–18. 10.1016/j.brat.2016.06.004. 28110678 10.1016/j.brat.2016.06.004

[25] Ter Huurne ED, de Haan HA, Postel MG, van der Palen J, van der Nagel JEL, el at. Web-based cognitive behavioral therapy for female patients with eating disorders: randomized controlled trial. J Med Internet Res. 2015;17:6. 10.2196/jmir.3946 10.2196/jmir.3946 PMC4526949 26088580

[26] Wu XY, Yin WQ, Sun HW, Yang SX, Li XY, et al. The association between disordered eating and health-related quality of life among children and adolescents: a systematic review of population-based studies. PLoS ONE. 2019;14(10):e0222777. 10.1371/journal.pone.0222777. 31584956 10.1371/journal.pone.0222777 PMC6777752

[27] Herpertz-Dahlman B, Wille N, Hölling H, Vloet TD, Ravens-Sieberer U. Disordered eating behaviour and attitudes, associated psychopathology and health-related quality of life: results of the BELLA study. Eur Child Adolesc Psych. 2008;17:82–91. 10.1007/s00787-008-1009-9. 10.1007/s00787-008-1009-9 19132307

[28] Calzo JP, Austin SB, Micali N. Sexual orientation disparities in eating disorder symptoms among adolescent boys and girls in the UK. Eur Child Adolesc Psych. 2018;27:1483–90. 10.1007/s00787-018-1145-9. 10.1007/s00787-018-1145-9 PMC6141356 29550905

[29] Grammer AC, Vazquez MM, Fitzsimmons-Craft EE, Fowler LA, Rackoff GN, et al. Characterizing eating disorder diagnosis and related outcomes by sexual orientation and gender identity in a national sample of college students. Eat Behav. 2021;42:e101528. 10.1016/j.eatbeh.2021.101528. 10.1016/j.eatbeh.2021.101528 PMC8380708 34049053

[30] Oflynn JL, Nowicki GP, Laveway K, Gordon AR, Rodgers RF. Toward inclusivity: A systematic review of the conceptualization of sexual minority status and associated eating disorder outcomes across two decades. Int J Eat Disord. 2023;56:350–65. 10.1002/eat.23830. 36321787 10.1002/eat.23830

[31] Cuijpers P, Auerbach RP, Benjet C, Bruffaerts R, Ebert D, Karyotaki E, Kessler RC. The world health organization world mental health college student initiative: an overview. Int J Methods Psychiatr Res. 2019;28:e1761. 10.1002/mpr.1761. 30614123 10.1002/mpr.1761 PMC6590455

[32] Andersson C, Bendtsen M, Lindfors P, Molander O, Lindner P, et al. Does the management of personal integrity information lead to differing participation rates and response patterns in mental health surveys with young adults? A three-armed methodological experiment. Int J Methods Psychiatr Res. 2021;30:e1891. 10.1002/mpr.1891. 34418224 10.1002/mpr.1891 PMC8633924

[33] Saunders JB, Aasland OG, Babor TF, de la Fuente JR, Grant M. Development of the alcohol use disorders identification test (AUDIT): WHO collaborative project on early detection of persons with harmful alcohol consumption II. Addiction. 1993;88:791–804. 10.1111/j.1360-0443.1993.tb02093.x 8329970 10.1111/j.1360-0443.1993.tb02093.x

[34] Government office/Regeringskansliet Follow-up of the government’s strategy of alcohol, drug, doping, and tobacco/Uppföljning av regeringens alkohol-, narkotika-, dopnings- och tobaksstrategi. The Ministry of Health and Social Affairs/Socialdepartementet. 2015;3: S2013.009

[35] Mortier P, Demyttenaere K, Auerbach RP, Cuijpers P, Green JG, Kiekens G, Kessler RC, et al. First onset of suicidal thoughts and behaviours in college. J Affect Disorders. 2017;207:291–9. 10.1016/j.jad.2016.09. 27741465 10.1016/j.jad.2016.09.033 PMC5460371

[36] Berman AH, Bendtsen M, Molander O, Lindfors P, Lindner P, et al. Compliance with recommendations limiting COVID-19 contagion among university students in Sweden: associations with self-reported symptoms, mental health and academic self-efficacy. Scand J Public Health. 2022;15:70–84. 10.1177/14034948211027824. 10.1177/14034948211027824 PMC8808007 34213359

[37] McGrath JJ, Al-Hamzawi A, Alonso J, Altwaijri Y, Andrade LH, Bromet EJ, Bruffaerts R, et al. Age of onset and cumulative risk of mental disorders: a cross-national analysis of population surveys from 29 countries. Lancet Psychiatry. 2023;10:668–81. 10.1016/S2215-0366(23)00193-1. 37531964 10.1016/S2215-0366(23)00193-1 PMC10529120

[38] Yu Z, Indelicato NA, Fuglestad P, Tan M, Bane L, Stice C. Sex differences in disordered eating and food addiction among college students. Appetite. 2018;129:12–8. 10.1016/j.appet.2018.06.028. 29935291 10.1016/j.appet.2018.06.028

[39] Zarakowski B, Giokaris D, Green O. Effects of the COVID-19 pandemic on university students’ mental health: a literature review. Cureus. 2024;16(2):e54032. 10.7759/cureus.54032. 38348205 10.7759/cureus.54032 PMC10859553

[40] Schegl S, Meule A, Favreau M, Voderholzer U. Bulimia nervosa in time of Covid 19 pandemic – results from an online survey of former inpatients. Eur Eat Disorders Rev. 2020;28:847–54. 10.1002/erv.2773. 10.1002/erv.2773 PMC7436773 32767858

[41] Birgergård A, Abbaspour A, Borg S, Clinton D, Forsén Mantilla E, Savva A, Termorshuizen JD, et al. Longitudinal experiences and impact of the COVID-19 pandemic among people with past or current eating disorders in Sweden. Eat Disorders. 2022;30:602–13. 10.1080/10640266.2021.1985286. 10.1080/10640266.2021.1985286 34634228

[42] Brausch AM, Perkins NM. Nonsuicidal self-injury and disordered eating: differences in acquired capability and suicide attempt severity. Psychiatry Res. 2018;266:72–8. 10.1016/j.psychres.2018.05.021. 29857291 10.1016/j.psychres.2018.05.021

[43] Cucchi A, Ryan D, Konstantakopoulos G, Stroumpa S, Kaçar AS, et al. Lifetime prevalence of non-suicidal self-injury in patients with eating disorders: a systematic review and meta-analysis. Psychol Med. 2016;46:1345–58. 10.1017/S0033291716000027. 26954514 10.1017/S0033291716000027

[44] Hasking P, Claes L. Transdiagnostic mechanisms involved in nonsuicidal self-injury, risky drinking and disordered eating: Impulsivity, emotion regulation and alexithymia. J Am Coll Health. 2020;68:603–9. 10.1080/07448481.2019.1583661. 30939103 10.1080/07448481.2019.1583661

[45] Kaplan A, Hutchinson A, Hooper S, Gwee K, Khaw D, Lola Valent L. Evaluation of an eating disorder screening and care pathway implementation in a general mental health private inpatient setting. J Eat Disorders. 2024;12:119. 10.1186/s40337-024-01077-x. 10.1186/s40337-024-01077-x PMC11334319 39160580

[46] Cao Z, Cini E, Pellegrini D, Fragkos KC. The association between sexual orientation and eating disorders-related eating behaviours in adolescents: a systematic review and meta-analysis. Eur Eat Disorders Rev. 2023;31:46–64. 10.1002/erv.2952. 10.1002/erv.2952 PMC10100331 36367345

[47] Croll J, Neumark-Sztainer D, Story M, Ireland M. Prevalence and risk and protective factors related to disordered eating behaviors among adolescents: relationship to gender and ethnicity. J Adolescent Health. 2002;31:166–75. 10.1016/s1054-139x(02)00368-3. 10.1016/s1054-139x(02)00368-3 12127387

[48] White A, Hingson R. The burden of alcohol use: excessive alcohol consumption and related consequences among college students. Alcohol Res - Curr Rev. 2013;35:201–18. 10.35946/arcr.v35.2.11 PMC3908712 24881329

[49] Kazdin AE, Fitzsimmons-Craft EE, Wilfley DE. Addressing critical gaps in the treatment of eating disorders. Int J Eat Disord. 2017;50:170–89. 10.1002/eat.22670. 28102908 10.1002/eat.22670 PMC6169314

[50] Rens E, Michielsen J, Dom G, Remmen R, Van den Broeck K. Clinically assessed and perceived unmet mental health needs, health care use and barriers to care for mental health problems in a Belgian general population sample. BMC Psychiatry. 2022;22:455. 10.1186/s12888-022-04094-9. 35799153 10.1186/s12888-022-04094-9 PMC9263045

[51] Monteleone AM, Barone E, Cascino G, Schmidt U, Gorwood P, Volpe U, Abbate-Daga G, et al. Pathways to eating disorder care: a European multicenter study. Eur Psychiatry. 2023. 10.1192/j.eurpsy.2023.23. 37092677 10.1192/j.eurpsy.2023.23 PMC10228357

[52] Hamilton A, Mitchison D, Basten C, Byrne S, Goldstein M, Hay P, Heruc G, et al. Understanding treatment delay: perceived barriers preventing treatment-seeking for eating disorders. Australian & New Zealand J Psychiatry. 2022;56:248–59. 10.1177/00048674211020102. 34250844 10.1177/00048674211020102

[53] Wagner B, Nagl M, Dölemeyer R, Klinitzke G, Steinig J, et al. Randomized controlled trial of an internet-based cognitive-behavioral treatment program for binge-eating disorder. Behav Ther. 2016;47:500–14. 10.1016/j.beth.2016.01.006 27423166 10.1016/j.beth.2016.01.006

[54] Wiberg A-C, Ghaderi A, Danielsson Broberg H, Safarzadeh K, Parling T, et al. Internet-based cognitive behavior therapy for eating disorders – Development and feasibility evaluation. Internet Intervent. 2022;30:e100570. 10.1016/j.invent.2022.100570. 10.1016/j.invent.2022.100570 PMC9468502 36110307

[55] Hedman-Lagerlöf E, Carlbring P, Svärdman F, Riper H, Cuijpers P, et al. Therapist-supported Internet-based cognitive behaviour therapy yields similar effects as face-to-face therapy for psychiatric and somatic disorders: an updated systematic review and meta-analysis. World Psychiatry. 2023;22:305–14. 10.17605/OSF.IO/VKPGB. 37159350 10.1002/wps.21088 PMC10168168

[56] Stice E, Telch CF, Rizvi SL. Development and validation of the eating disorder diagnostic scale: a brief self-report measure of anorexia, bulimia, and binge-eating disorder. Psychol Assessment. 2000;12:123–31. 10.1037/1040-3590.12.2.123 10.1037//1040-3590.12.2.123 10887758

[57] Penninx BWJH, Benros ME, Klein RS, Vinkers CH. How COVID-19 shaped mental health: from infection to pandemic effect. Nature Med. 2022;28:2027–37. 10.1038/s41591-022-02028-2. 36192553 10.1038/s41591-022-02028-2 PMC9711928

