# Supplementary material for: Eating disorders and psychiatric comorbidity among first-year university students in Sweden: Prevalence and risk factors
Source: J Eat Disord. 2025 Mar 20;13:52. doi: 10.1186/s40337-025-01230-0 (PMC11924712; doi:10.1186/s40337-025-01230-0)
Supplement: Supplementary file 1 — Additional file1 (DOCX 20 KB) [file 40337_2025_1230_MOESM1_ESM.docx]

Table S1. Comparisons between consenting survey-completers and non-completers on sex at birth (chi2-test), age, self-rated physical and mental health (independent sample t-tests with a 0.05 confidence intervals).

|  | Completers  (n=3425) | Non-completers  (n=1749) | *df* | *Chi-2/t* | *p* |
| --- | --- | --- | --- | --- | --- |
| Sex (%)   - Male - Female | 29  71 | 33  67 | 2 | 20.94 | <0.001 |
| Age M (SD) | 23(12) | 20(22) | 5168 | 7.3 | <0.001 |
| Physical health* | 2.5(5.0) | 1.8(9.8) | 5137 | 3.4 | 0.001 |
| Mental health* | 3.0(4.1) | 2.6(8.6) | 5138 | 2.7 | 0.007 |

*Higher numbers indicate worse physical and mental health
