# Supplementary material for: Eating disorders and psychiatric comorbidity among first-year university students in Sweden: Prevalence and risk factors
Source: J Eat Disord. 2025 Mar 20;13:52. doi: 10.1186/s40337-025-01230-0 (PMC11924712; doi:10.1186/s40337-025-01230-0)
Supplement: Supplementary file 2 — Additional file2 (DOCX 23 KB) [file 40337_2025_1230_MOESM2_ESM.docx]

Table 5. Result of multinomial logistic regression, age and sex at birth included, with three groups: A) eating disorders (ED) with or without psychiatric comorbidity, B) Psychiatric disorders without ED comorbidity, and C) No psychiatric disorders. Group B is the reference in the model.

|  | b(SE) | Odds ratio | CI [95%]  Lower | Upper |
| --- | --- | --- | --- | --- |
| **Group A: ED** **with or without psychiatric comorbidity^a^** |  |  |  |  |
| Intercept | -.091(.229) |  |  |  |
| Age | -.013(.009) | .987 | .970 | 1.004 |
| Sex at birth | -1.034(.110)*** | .355 | .286 | .441 |
| Physical health, self-rated | -.184(.102) | .832 | .681 | 1.017 |
| Mental health, self-rated | -.167(.093) | .846 | .705 | 1.015 |
| Hazardous drinking | .409(.095)*** | 1.506 | 1.250 | 1.814 |
| Sexual orientation |  |  |  |  |
| - Gay or Lesbian | -.162(.236) | 1.176 | .741 | 1.866 |
| - Bisexual | .224(.121) | 1.251 | .986 | 1.588 |
| - Asexual | .421(.349) | 1.524 | .768 | 3.022 |
| - Do not know | - .025(.178) | .976 | .689 | 1.382 |
| - Other | .311(.252) | 1.365 | .833 | 2.237 |
| Treatment, lifetime |  |  |  |  |
| - Psychological | .056(.104) | 1.058 | .863 | 1.297 |
| - Medical | .334(.115)** | 1.397 | 1.115 | 1.749 |
| - Other | .482(.149)** | 1.620 | 1.209 | 2.171 |
| **Group C: No psychiatric disorders** |  |  |  |  |
| Intercept | -2.714(.302)*** |  |  |  |
| Age | .021(.009)* | 1.021 | 1.003 | 1.041 |
| Sex at birth | -.007(.113) | .933 | . 796 | 1.239 |
| Physical health, self-rated | .732(.183)*** | 2.079 | 1.453 | 2.975 |
| Mental health, self-rated | 1.608(.162)*** | 4.992 | 3.635 | 6.855 |
| Hazardous drinking | -.350(.130)** | .705 | .546 | .910 |
| Sexual orientation |  |  |  |  |
| - Gay or Lesbian | -.608(.434) | .544 | .232 | 1.275 |
| - Bisexual | -.807(.245)** | .446 | .276 | .722 |
| - Asexual | -1.217(.776) | .296 | .065 | 1.356 |
| - Do not know | -.757(.320)* | .469 | .251 | .878 |
| - Other | -.706(.477) | .493 | .194 | 1.258 |
| Treatment, ever |  |  |  |  |
| - Psychological | -1.294(.163)*** | .274 | .199 | .377 |
| - Medical | -.912(.262)*** | .402 | .240 | .672 |
| - Other | -.479(.293) | .620 | .349 | 1.101 |

^a^Students with only ED were included in this group.

*p < 0.05, **p < 0.01, ***p < 0.001
